# Endoscopic severe mucosal atrophy indicates the presence of gastric cancer after *Helicobacter pylori* eradication -analysis based on the Kyoto classification

**DOI:** 10.1186/s12876-020-01375-z

**Published:** 2020-07-20

**Authors:** Akiko Ohno, Jun Miyoshi, Atsushi Kato, Naohiko Miyamoto, Takahito Yatagai, Yu Hada, Mitsunori Kusuhara, Yoko Jimbo, Yosuke Ida, Kengo Tokunaga, Susumu Okamoto, Tadakazu Hisamatsu

**Affiliations:** 1 Department of Gastroenterology and Hepatology, Kyorin University School of Medicine, 6-20-2 Shinkawa, Mitaka-shi, Tokyo, 181-8611 Japan; 2 Department of General Medicine, Kyorin University School of Medicine, 6-20-2 Shinkawa, Mitaka-shi, Tokyo, 181-8611 Japan

**Keywords:** Stomach neoplasms, Helicobacter pylori, Atrophy, Endoscopy

## Abstract

**Background:**

Gastric cancer after *Helicobacter pylori* (HP) eradication is a crucial clinical challenge today as HP eradication therapy is widely performed. Detecting gastric cancer after HP eradication tends to be difficult with normal white-light endoscopy. In the present study, we aimed to identify easily-evaluated endoscopic findings that indicate the presence of gastric cancer after HP eradication so that endoscopists can consider additional detailed examinations at the site.

**Methods:**

We analyzed the endoscopic images of 43 patients who underwent endoscopic submucosal dissection for early gastric cancer after HP eradication and 119 patients with an HP eradication history who underwent esophagogastroduodenoscopy for a medical checkup. Endoscopic findings were evaluated according to the Kyoto classification of gastritis (atrophy, intestinal metaplasia, enlarged folds, nodularity, and diffuse redness) and map-like redness.

**Results:**

Patients with gastric cancer had significantly higher total Kyoto risk scores; more atrophy, intestinal metaplasia, and diffuse redness; and a significantly higher prevalence of map-like redness compared with those without gastric cancer, in the univariate analyses. We used logistic regression analysis with forward selection based on the likelihood ratio to develop a model using atrophy and diffuse redness. Receiver operating characteristic analysis showed that a score of A2 in the Kyoto classification of gastritis (open-type atrophic pattern in the Kimura–Takemoto classification) was an endoscopic marker for the presence of post-HP-eradication gastric cancer.

**Conclusions:**

Endoscopic severe gastric mucosal atrophy is useful to screen patients for gastric cancer after HP eradication.

## Background

**Fig. 1:**
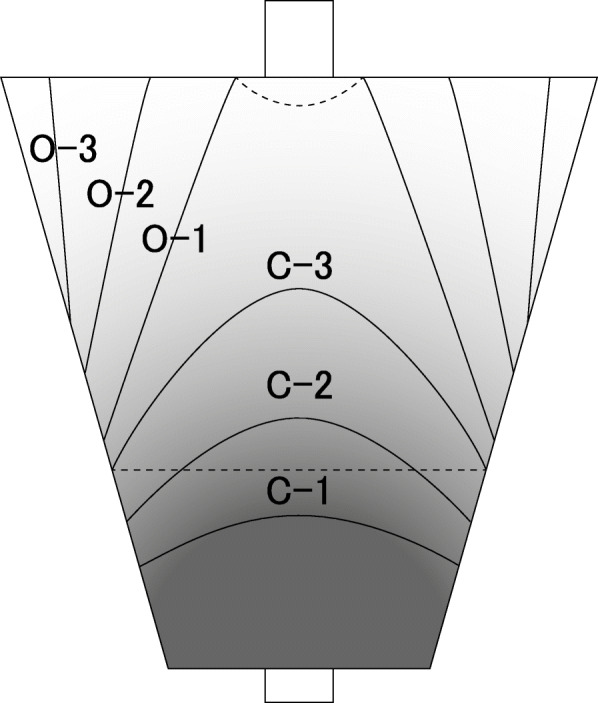
Kimura-Takemoto classification. Depending on the atrophic border, it is classified into a closed type that does not exceed the cardia (C-1, 2, and 3) and an open type that extends beyond the cardia to a greater curvature (O-1, 2, and 3)

Gastric cancer is the fifth leading cancer and its incidence is estimated to be more than 1 million patients, in 2018 [1]. *Helicobacter pylori* (HP) is a class-I carcinogen for gastric cancer [2]. Chronic HP infection causes atrophic gastritis and intestinal metaplasia leading to dysplasia and the development of gastric cancer [3, 4]. HP eradication has efficacy against the development of gastric cancer [5, 6]. These facts underscore the importance of diagnosing *HP* infection and assessing the risk of gastric cancer in HP-positive patients. The Kimura–Takemoto classification system has long been used to describe atrophic gastritis [7] (Fig. 1). The Sydney system was proposed and revised in 1990 and in 1996 [8], respectively, for the endoscopic assessment of gastritis regarding HP infection [9]. However, this system includes not only endoscopic but also pathological findings, and some endoscopic findings are difficult to objectively evaluate. More importantly, this system is not designed for assessing the risk of gastric cancer. Therefore, a scoring system based on both the HP infection diagnosis and the risk assessment of gastric cancer was needed. To address this need, the Kyoto classification of gastritis was developed in 2015 [10]. In this scoring system, five endoscopic findings (atrophy, intestinal metaplasia, gastric fold hypertrophy, nodularity, diffuse redness) related to HP infection are scored to estimate the risk for gastric cancer. With this scoring system, Sugimoto et al. reported that the scores for intestinal metaplasia and atrophy are useful for screening high-risk patients for gastric cancer among HP-positive patients with chronic gastritis [11]. However, it must be noted that the Kyoto classification was originally designed for HP-positive patients and not for assessing the risk of gastric cancer after HP eradication.

While HP eradication therapy is now widely performed, and its success rate is almost 90% with progress in eradication regimens [12, 13], gastric cancer that develops after HP eradication is emerging as a new clinical challenge [14, 15]. Detecting gastric cancer endoscopically after successful HP eradication is thought to be more difficult than HP-positive gastric cancer because of the histological effects of eradication [16]. Kobayashi et al. reported that gastric cancer after eradication tends to look similar to gastritis because the cancer can be covered with normal differentiated epithelium. The authors estimated that this morphological characteristic occurs in up to 44% of patients with gastric cancer after HP eradication [17]. Therefore, to develop an efficient surveillance method to screen high-risk patients, it is crucial to identify the endoscopic findings of the gastric mucosa that alert endoscopists to the presence of gastric cancer after HP eradication.

In the present study, we aimed to identify the endoscopic findings according to the Kyoto classification that provide an indication of the presence of gastric cancer in patients after HP eradication.

## Methods

### Study design

In this retrospective cross-sectional study, we investigated the clinical database of Kyorin University Hospital. Gastric endoscopic submucosal dissection was performed for 209 patients with early gastric cancer in Kyorin University Hospital from April 2013 to July 2018. Gastric cancers were diagnosed by endoscopy and confirmed histologically by registered pathologists at Kyorin University Hospital. Among these 209 patients, 61 patients had an HP eradication history. To assess the development of gastric cancer after HP eradication, we analyzed data for 43 patients who underwent HP eradication more than 1 year before endoscopic submucosal dissection (ESD). The success of HP eradication was determined by a urea breath test or a fecal HP antigen test. The age range of these patients was 58–87 years. To form a control group, we screened patients undergoing a medical checkup that included esophagogastroduodenoscopy (EGD) in Kyorin University Hospital from February 2018 to July 2018 and who had a history of HP eradication more than 1 year before EGD. Among the potential controls, we excluded patients with a history of gastric cancer and gastrectomy, while included the subjects at the age of 58–87 matching the age of ESD group. A final 162 patients (43 patients with gastric cancer and 119 patients without gastric cancer) were included in the present study. The clinical data of these subjects including sex, age, duration after HP eradication, and smoking history was investigated. Smoking history was assessed with Brinkman index which is numbers of cigarettes smoked per day-years, given the association between smoking and gastric cancer [18].

### Endoscopic assessment

The EGD findings were reviewed by 8 experienced endoscopists and they scored the five endoscopic findings according to the Kyoto classification of gastritis (Table 1) [10]. Multiple endoscopists scored together for each subject. Endoscopic images by white light but not image-enhanced endoscopy were used in this study. The scored endoscopic findings were: (1) atrophy: A0 = no atrophy (C0); C1, A1 = C2 and C3; and A2 = O1, O2, O3 (C0–C3 and O1–O3 are atrophic pattern in the Kimura–Takemoto classification); (2) intestinal metaplasia: IM0 = negative, IM1 = antrum area, and IM2 = antrum to gastric body; (3) enlarged folds: H0 = the width of the folds are ≤4 mm under observation with a sufficient amount of air, H1 = the width of the folds are ≥5 mm; (4) nodularity: N0 = negative, N1 = positive; and (5) diffuse redness: DR0 = visible regular arrangement of collecting venules (RAC), DR1 = partially visible RAC, DR2 = disappearance of RAC. Additionally, because map-like redness is a characteristic after HP eradication [19, 20], we assessed the presence or absence of map-like redness.

**Table 1:** Grading Scores of Cancer in the Kyoto Classification of Gastritis

Elements	Score		
A: Gastric mucosal atrophy	0	None	C0-C1(according to Kimura-Takemoto classification)
	1	Mild	C2-C3
	2	Severe	O1-O3
IM: Intestinal metaplasia	0	None	None
	1	Mild	Within the antrum
	2	Severe	Up to the Corpus
H: Hypertrophy of gastric fold	0	Negative	≦4 mm
	1	Positive	>5 mm
N: Nodularity	0	Negative	Negative
	1	Positive	Positive
D: Diffuse redness	0	None	None
	1	Mild	Mild translucency of collecting venules in the body
	2	Sever	Severe translucency of collecting venules in the body

### Statistical analysis

Fisher’s exact test was performed to compare the sex ratio and the presence of map-like redness between groups with/without gastric cancer. We used the Mann–Whitney *U* test to compare age, duration after HP eradication, Brinkman’s index, and the scores for the endoscopic findings between the groups. Logistic regression analysis with forward selection based on the likelihood ratio was performed for a multivariate analysis of the endoscopic findings. We used a receiver operating characteristic analysis to assess the usefulness of the regression equation and to determine the threshold. The criterion of statistical significance was set at *P* <  0.05. IBM SPSS Statistics (ver. 24) (IBM Corp., NY) and GraphPad Prism (ver.8.1.2) (GraphPad Software, San Diego, CA) were used for logistic regression analysis and for other statistical analyses, respectively.

## Results

### Patients’ characteristics

**Fig. 2:**
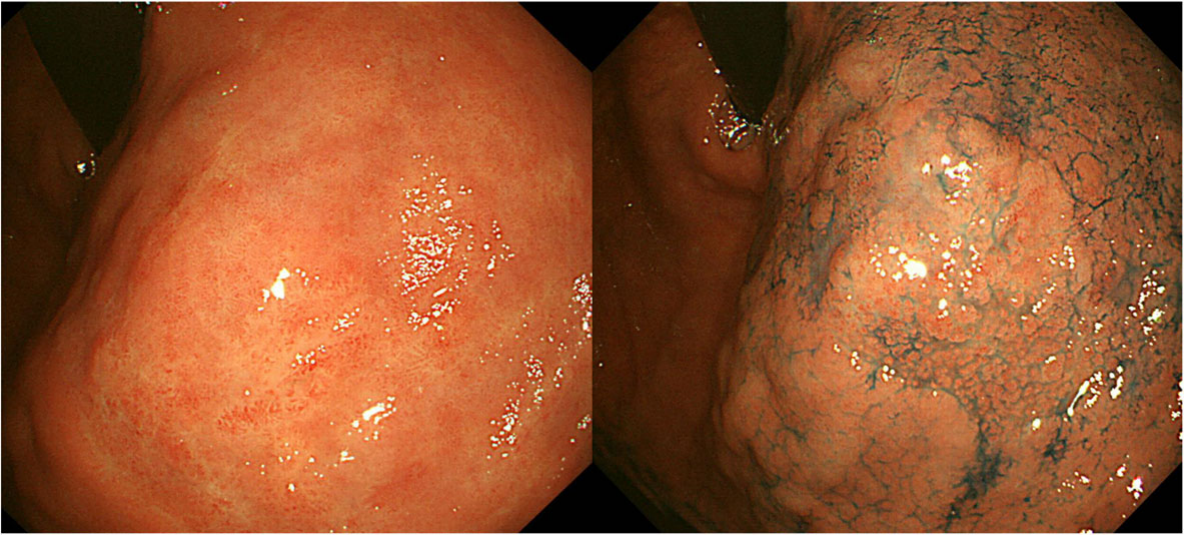
Representative early gastric cancer after HP eradication. A representative case of 25 mm 0-IIb lesion of the upper gastric body at 8 years after HP eradication is presented. The atrophy of background gastric mucosa was A2 type in Kyoto classification. The left and right panels show the same lesion with white-light endoscopy and chromo endoscopy, respectively

Forty-three patients with gastric cancer and 119 patients without gastric cancer who achieved HP eradication were analyzed in the present study. Among the gastric cancer patients, 90.7% of the subjects (39 out of 43 cases) had EGD as a regular check-up without apparent gastric symptoms. Meanwhile, 81.5% of the control group (97 out of 119 cases) had EGD in the study period as an annual health check-up at our facility without any symptoms, 2.5% (3 subjects) as the first-time health check-up. Patients’ characteristics in the groups with/without gastric cancer are shown in Table 2. ESD was successfully performed for the patients with early gastric cancer (Supplementary Table 1). All early gastric cancers detected in the present study were located in the atrophic area. Representative endoscopic findings are shown in Fig. 2. The sex ratio was significantly different between the groups (*P* = 0.0006). There was no significant difference in the duration after HP eradication between the groups, and patients without gastric cancer were younger than those with gastric cancer (*P* = 0.0305). There was no significant difference in Brinkman’s index between the groups.

**Table 2:** Characteristics of Patients

	Gastric cancer (+)	Gastric cancer (−)	*P* value
Number	43	119	–
Sex (F/M)	5/38	47/72	0.0006^a^
Age (years old, median, range)	72 (58–87)	69 (58–86)	0.0305^b^
Duration after HP eradication (months, mean ± SEM)	66.88 ± 7.170	73.79 ± 5.221	n.s.^b^
Brinkman Index (mean ± SEM)^c^	405.4 ± 89.33	264.2 ± 34.88	n.s.^b^

^a^Fisher’s exact test, ^b^Mann-Whitney *U* test

^c^The smoking history was not provided by one subject in the gastric cancer (+) group and two subjects in the gastric cancer (−) group

### Differences in gastric mucosal endoscopic findings between patients with and without gastric cancer after HP eradication

The risk score according to the Kyoto classification of gastritis (total Kyoto risk sore) and scores for each endoscopic finding (atrophy, intestinal metaplasia, enlarged folds, nodularity, and diffuse redness) were compared between the groups with/without gastric cancer by univariate analyses (Table 3). The group with gastric cancer had the higher total Kyoto risk score (4.163 ± 0.1049) compared with the group without gastric cancer (2.361 ± 0.1263) (*P* <  0.0001). Among the five endoscopic findings, scores for atrophy (2.000 ± 0.000 vs. 1.496 ± 0.04755; *P* <  0.0001), intestinal metaplasia (1.814 ± 0.08323 vs. 1.076 ± 0.08619; *P* <  0.0001), and diffuse redness (0.2326 ± 0.08040 vs. 0.04167 ± 0.01832; *P* = 0.0027) were significantly higher in patients with gastric cancer compared with those without cancer, respectively. The prevalence of map-like redness was significantly higher in patients with gastric cancer than in patients without gastric cancer (60.5% vs. 31.1%, respectively; *P* = 0.0010). Next, we performed a logistic regression analysis with forward selection based on the likelihood ratio using the endoscopic findings that showed significant differences in the univariate analyses, namely, atrophy, intestinal metaplasia, diffuse redness, and map-like redness. As shown in Table 4, we developed a model with atrophy and diffuse redness (Hosmer–Lemeshow test: *P* = 1.000). The odds ratios of atrophy (*p* = 0.997) and diffuse redness (*P* = 0.035) were 1,013,681,190.83195 and 3.988, respectively.

**Table 3:** Univariate analyses for endoscopic findings

	Gastric cancer (+)	Gastric cancer (−)	*P* value
Kyoto risk score (total)	4.163 ± 0.105	2.681 ± 0.126	< 0.0001^b^
-Atrophy	2.000 ± 0.000	1.496 ± 0.048	< 0.0001^b^
-Intestinal metaplasia	1.814 ± 0.083	1.076 ± 0.086	< 0.0001^b^
-Enlarged fold	0.116 ± 0.049	0.050 ± 0.020	0.1626^b^
-Nodularity gastritis	0.000 ± 0.000	0.008 ± 0.008	> 0.9999^b^
-Diffuse redness	0.233 ± 0.080	0.042 ± 0.018	0.0027^b^
Map-like redness (+/−)	26/17	37/82	0.0010^a^

The scoring data is presented as mean ± SEM

^a^Fisher’s exact test, ^b^Mann-Whitney *U* test

**Table 4:** Logistic regression analysis for endoscopic findings (forward selection based on likelihood ratio)

	Regression coefficient	Standard error	Waldχ^2^ value	*P* value	Odds ratio	95% CI of Odds ratio	
						Lower	Upper
Atrophy	20.737	5101.770	0.000	0.997	1,013,681,190.832	0.000	
Diffuse redness	1.383	0.655	4.467	0.035	3.988	1.106	14.386
Constant	−41.970	10,203.539	0.000	0.997	0.000		

Model χ^2^ test: *P* <  0.001

Hosmer-Lemeshow test: *P* = 1.000

Percentage of correct classifications: 76.5%

### Gastric mucosal atrophy indicates the presence of gastric cancer after HP eradication

Based on the regression equation (Table 4), the probability of the presence of gastric cancer was calculated for all 162 patients (Supplementary Table 1). We evaluated the predictive performance of this calculated probability for the presence of gastric cancer using a receiver operating characteristic curve analysis. The area under the curve was 0.7828 (95% confidence interval (CI): 0.7131–0.8524), and Youden’s index suggested the threshold as 0.1892 (Table 5). With the threshold set at 0.1892, sensitivity was 100.0% (95% CI: 91.8–100.0%), specificity was 49.58% (95% CI: 40.75–58.43%), positive predictive value was 41.74%, and negative predictive value was 100.0% (Table 6). There are nine possible combinations of the atrophy score (0–2) and diffuse redness score (0–2), and the calculated probability was larger than 0.1892 only when the atrophy score equaled 2, regardless of the diffuse redness score (Table 7). These results indicated that severe gastric mucosal atrophy (atrophy score = 2) is an endoscopic marker to predict the presence of gastric cancer.

**Table 5:** ROC analysis between gastric cancer and calculated probability

ROC curve					
AUC	95% CI	*P* value			
0.7828	0.7131–0.8524	< 0.0001			
ROC sensitivity, specificity and likelihood					
	Sensitivity (%)	95% CI	Specificity (%)	95% CI	Likelihood ratio
> 0.1892	100.0	91.80–100.0%	49.58	40.75–58.43%	1.983

*ROC* receiver operating characteristic, *CI* confidence interval 
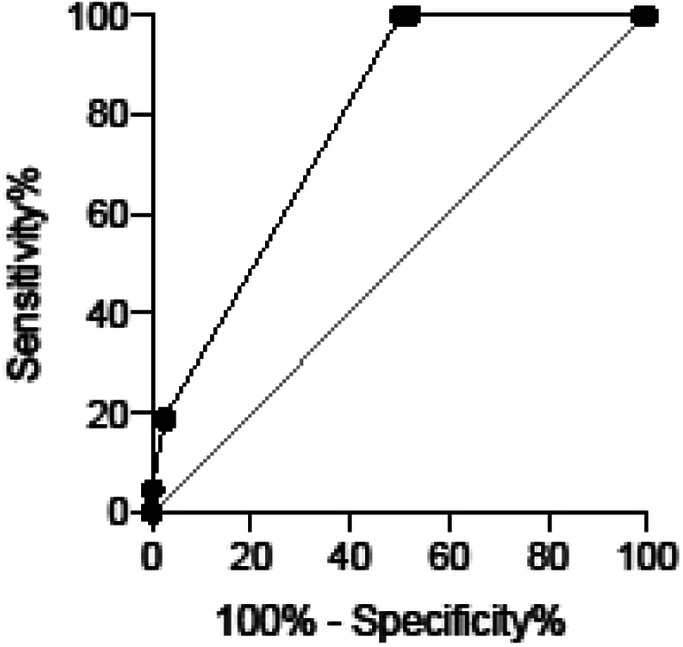

**Table 6:** Contingency table for gastric cancer and proposed threshold of calculated probability

		Calculated probability	
		*P* > 0.1892	*P* < 0.1892
Gastric cancer	(+)	43	0
	(−)	60	59

Sensitivity: 100.0%

Specificity: 49.58%

Positive predictive value: 41.74%

Negative predictive value: 100.0%

**Table 7:** Chart for calculated probability of gastric cancer

Atrophy score	Diffuse redness score	Calculated probability
2	2	0.906361788
2	1	0.70827069
2	0	0.378481143
1	2	9.54737E-09
1	1	2.39472E-09
1	0	6.00655E-10
0	2	9.41714E-18
0	1	2.36205E-18
0	0	5.92462E-19

## Discussion

In the present study, we examined five endoscopic findings according to the Kyoto classification of gastritis and the finding of map-like redness to identify the endoscopic makers for the presence of gastric cancer after HP eradication. Given the difficulty in detecting early gastric cancer in HP-eradicated stomachs [17], it is important to determine which markers indicate a high risk. We demonstrated that endoscopic severe gastric atrophy (score A2 in the Kyoto classification of gastritis, open type atrophy in the Kimura–Takemoto classification) is a crucial clue to suspect the presence of gastric cancer in patients after HP eradication. This finding is clinically important and useful because open type atrophy is easily detected by checking the atrophic border, and the diagnosis does not require biopsy. Our finding suggests that a detailed observation (e.g., image-enhanced endoscopy, chromoendoscopy, and more biopsies for suspected lesions) is recommended during EGD for HP-eradicated patients with A2 atrophy. Interestingly, a recent study also observed that the advanced atrophy can be a risk factor for gastric cancer after HP eradication [21].

HP infection is thought to occur in early childhood when gastric acid is weak and HP can survive [22, 23]. After infection, gastric inflammation and mucosal atrophy gradually expand to the gastric body. Several studies showed that HP eradication improves gastric mucosal atrophy [24, 25]. However, even after successful HP eradication, improvement in gastric mucosal lesions may take a long time. Toyokawa et al. reported that improvement in atrophic gastritis requires approximately 9 years [25]. Given the infection period from early childhood, older patients can be infected with HP for a longer time and have a wider range of gastric mucosal atrophy (i.e., a higher score for endoscopic atrophy) compared with younger patients. In our study, the group with gastric cancer was significantly older than the control group, while there was no significant difference in the duration after HP eradication between the groups. This difference in the period of HP infection may affect the extent of atrophy that leads to the development of gastric cancer. Meanwhile, since HP eradication improves gastric mucosal atrophy [25], early HP eradication before atrophy become severe can prevent further gastric atrophy progression. Taken together, our findings suggest that HP eradication when atrophy is not severe may reduce the risk of gastric cancer development later in life. Shichijo et al. reported that intestinal metaplasia histology and endoscopic mucosal atrophy can predict the future development of gastric cancer after HP eradication [26]. The authors used the Kyoto classification of gastritis scoring system to assess gastric mucosal atrophy and showed that patients with an A2 score are at increased risk for gastric cancer. Considering our findings, strict EGD follow-ups with the presence of gastric cancer in mind are recommended for patients with an A2 score after HP eradication.

There are several limitations in the present study. First, this was a single-center study, and the number of patients was limited. Although multiple endoscopists were involved in this study, a larger multiple-center study is needed to validate our findings. In addition, the reviewing endoscopic findings for cases with/without gastric cancer could not be perfectly blinded because gastric cancer was in some of images in the examination for subjects with gastric cancer. Second, gastric cancer in this study included only early gastric cancer satisfying the indications for endoscopic resection but not advanced cancers requiring surgery or other therapeutic options. However, the rationale of the study design was (1) advanced cancer could affect the background gastric mucosa (e.g., inflammation, lymphatic stasis), (2) detecting early difficult-to-find cancer is a clinical challenge, and (3) all advanced cancer must pass the early stage. Third, HP reinfection was not tested before endoscopy in this study. Since reinfection rate of HP after eradication is very low in Japan [27], it is uncommon to check HP reinfection in clinical settings in Japan.

While this study showed the current risk of gastric cancer, a further longitudinal study in the group without gastric cancer is needed to investigate (1) whether endoscopic severe atrophy is a major risk factor for future carcinogenesis after HP eradication and (2) whether the long-term risk can be reduced once atrophy improves.

## Conclusion

Endoscopic severe gastric mucosal atrophy (A2 score in the Kyoto classification of gastritis, O1–O3 score in the Kimura–Takemoto classification) is easy to assess and useful to screen patients with gastric cancer after HP eradication.

## Supplementary information

**Additional file 1: Supplementary Table 1**. Early gastric cancers in the 43 subjects with ESD.

**Additional file 2: Supplementary Table 2**. Calculated probability of gastric cancer in all subjects.

## Data Availability

The datasets used and analyzed during this study are available from the corresponding author on reasonable request.

## Abbreviations

EGD: Esophagogastroduodenoscopy
ESD: Endoscopic submucosal dissection
HP: *Helicobacter pylori*
RAC: Regular arrangement of collecting venules
